# The Value of Songs for Teaching and Learning Cardiopulmonary Resuscitation (CPR) Competencies: A Systematic Review

**DOI:** 10.7759/cureus.15053

**Published:** 2021-05-16

**Authors:** Jeffrey L Pellegrino, Jennifer Vance, Nicholas Asselin

**Affiliations:** 1 School of Disaster Sciences and Emergency Services, University of Akron, Akron, USA; 2 Nursing, Aultman College of Nursing and Health Sciences, Canton, USA; 3 Emergency Medicine, Brown University, Providence, USA

**Keywords:** teaching aids, learning, bpm, first aid, education, cpr, resuscitation, songs, mental metronome, compression

## Abstract

We sought to summarize, in a systematic review, the effectiveness of songs to support learning, performance, and recall of quality characteristics of cardiopulmonary resuscitation (CPR) compression rate, and depth. We systematically reviewed the literature from eight academic indexes from the fields of medicine, nursing, allied health, and education, from 2014 to 2020 to identify studies that evaluated an intervention of song use during CPR training against control and reported outcomes of compression rate and depth.

There were 185 studies initially identified for review, eight met criteria for inclusion and analysis. For the critical outcome of compression depth, a pooled song group (n=446) when compared to a non-song group (n=443) demonstrated higher odds of being in the recommended range (OR 3.47). All studies, however, performed an average compression depth shallower than recommended guidelines in each arm. The available literature, we found, utilized heterogenous methodology and was at high risk of bias. When pooled, there were trends towards improved CPR metric performance in groups who were exposed to songs during treatment, though this only reached significance when groups were tested at >30 days from initial exposure. Findings of lower compression rates in the song groups suggest that song selection should favor beats per minute closer to the midpoint of the 100-120 ideal range to allow for variation when used as mental metronomes.

## Introduction and background

The benefits of early and proper cardiopulmonary resuscitation (CPR) for a person in sudden cardiac arrest prior to Emergency Medical Services (EMS) arrival improve the odds of survival to release from a hospital two to three-fold from those not receiving lay responder CPR [1]. As health practitioners, we are in the position to not only provide quality evidence-based CPR but prepare and or educate individuals and the public to respond alike. However, the public does not have the same motivation, access to continued training, or legal mandate to be proficient in basic life support (BLS) skills of CPR. Response by laypersons (not-medically trained) is a complicated set of competencies that involves knowledge, skills, and behaviors specific to cardiac arrest, wrapped in a set of the attitude of willingness, self-efficacy, and confidence in one’s ability to save a life [1-6]. Thus far, efforts to improve lay response in cardiac arrest have included mass training events [7], training “prescriptions” for high-risk groups [8], technology-driven solutions to alert trained responders to emergencies [9], and the development of telecommunicator CPR for just in time training [10]. These efforts assume that there is a basic pedagogy for effective CPR.

The International Liaison Committee on Resuscitation provides a consensus on science and treatment recommendations that the American Heart Association and American Red Cross use to identify the components of effective chest compressions and their significance to survival [11,12]. Effectiveness was a combination of correct hand position, compression rate, compression depth, and chest wall recoil. Making these CPR competencies accessible to BLS learners in training ideally translates to competent actions in emergency situations, which motivated us to identify evidence of effective and efficient educational tools to use across training modalities.

A perennial question from the field, both the public and instructors, to the American Red Cross’ national Scientific Advisory Council, concerns the efficacy of using music to teach or associate the current 100-120 chest compressions per minute (CPM) rate. Public health messaging, training programs, and individual educators have been promoting or using songs like “Stayin’ Alive” [13] to provide a cadence for learning and inspiring recall for the rate of compressions. Traditionally, facilitated/coached practices supplement audio/video-guided demonstrations to provide guidance and feedback during the practice of compressions [14-22]. Teaching initial compression competencies as well as supporting recall challenges CPR educators to understand the physical, social, and technological backgrounds of learners. The longitudinal recall is important because of the potentially long duration before an emergency might require action. Compression-only CPR is now taught to simplify and reduce barriers to the action of lay responders, which makes teaching compressions effectively a priority [23,24]. Songs, as a classroom tool, are an extension of metronome type guidance in a relatively inexpensive and potentially socially connected manner. They may also be supportive of recall and mentally guide compressions when technology is not available to provide active or passive feedback (e.g., automated external defibrillator [AED] prompts, smartphone apps, or telecommunicators).

Opportunities to engage music as a device for initially learning the CPR compression rate, assist in recall of the rate, and pace actual compressions may offer training organizations and individual educators new tools to engage learners and meet their educational and health outcomes. The value of songs in this education process is the subject of this systematic review of existing literature. The research question for this review: do songs (or does music) improve the learning and/or performance of CPR compressions?

## Review

This review was approved by the Scientific Advisory Council of the American Red Cross as an update to an internal 2014 review of this question.

Eligibility and inclusion criteria

The participants in studies were individuals learning or demonstrating CPR chest compression competencies, there was no exclusion by age. Each study needed to have an educational intervention that included a song to teach, coach, or assist with a recall of CPR compression rate. Studies also needed a comparison or control group. We looked to identify the critical outcomes of compression rate and compression depth at zero days post-training, 1-29 days, and >30 days post-training. Important outcomes included chest compression rate, depth, fraction, learner self-efficacy, and learner confidence. We excluded studies that were not published original research articles on randomized and non-randomized education interventional studies or observational studies with a comparator group. Although we were most interested in lay responder outcomes, we did not exclude health professionals because outcomes could be potentially extrapolated. Publication dates were between 2014 and 2020, due to a previous review of the literature by the Red Cross from 2010 to 2014. Abstracts needed to be in English for initial inclusion.

Information sources

The following electronic databases were searched due to their coverage of health, education, and psychometric topics: PubMed, Embase, CINAHL, ERIC, Psychology and Behavioral Sciences Collection, PsycARTICLES, Health Source: Nursing/Academic Edition/Health Source: Consumer Edition, and The Networked Digital Library of Theses and Dissertations (NDLTD). See Appendix A for search strategy strings, developed with the Health Science Library of the New York Medical College. We searched reference lists from included studies and reviewed articles for additional papers.

Study Selection

We used predefined criteria for what citations to include, which Author A and Author B independently applied for initial screening, utilizing Rayyan QCRI web software [25]. Titles and abstracts with clearly unrelated content were “excluded” from further analysis. Citations marked as “include” or “maybe” by either of the reviewers were included in the next level of review. Disagreements at the full article stage were resolved through consensus with the assistance of a third reviewer (Author C). A record was kept of all studies excluded at the full-text stage, along with the reason.

Data Collection Process

Author B extracted the intervention characteristics and data of interest by hand from the studies and Author A checked the extracted data, and any discrepancies were resolved through consensus utilizing Review Manager (RevMan) 5.3.

**Table 1:** Characteristics of Included Studies The same study, comparator though is to metronome guidance v. no song NA = Not Applicable; nr = not reported; cpm = compressions per minute; RCT = Randomized Control Study

Author	Year	Continent	Study Type	Song	Song Beats Per Minute	Medical trained Participants	Song Instruction/ coaching (active feedback)	Song Listening while performing	Target Compression Rate/ Context	Duration of test (Min)	Intervention/ Control size	Outcomes	Recall
Roach et al. [26]	2014	N. America	RCT	Songs	average 113	+	+	-	100-120 cpm	1	T_0_: 32/32 T_>30_: 29/ 31	Dichotomous In Range Mean CPM Knowledge Confidence	0 days/ 42 days
Roehr et al. [27]	2014	Australia	Randomized by song	Songs	average 115	+	-	+	90 cpm: 30 ventilations	nr	6 songs: no-song	Rate of compressions with ventilations	NA
Hafner et al. [28]	2015	N. America	RCT	Stayin' Alive	103	-	+	-	100-120 cpm	2	T_0_: 50/46 T_>30_: 46/42	Dichotomous In Range Mean CPM Mean Compression Depth	0 days/ >42 days
Hong et al. [29]	2016	Asia	RCT	Men are Ships, Women are Ports	100	nr	+	-	100-120 cpm	2	T_0_: 68/61 T_>30_: 68/61	Dichotomous In Range Mean Compression Depth Ratio of Correct Compression Depth Correct Hand Position	0/80 days
Leung [30]	2016	Asia	RCT	Stayin' Alive	104	+	+	+	≥100 cpm	2	T_0_: 17/20 T_1-29_: 17/20	Dichotomous In Range Mean CPM Mean Compression Depth Percent of Correct Depth Complete Recoil No Flow Time	0 days/ ≈7 days
Tastan et al. [31]	2016	Europe/Asia	RCT	modified Stayin' Alive	103	-	+	-	100-120 cpm	1	T_1-29_:39/38 T_>30_: 38/37	Dichotomous In Range Mean CPM	1day/42 days
Kim et al. [32]	2017	Asia	RCT	Songs [v. control]	average 102	+	+	nr	≥100 cpm	1	T_1-29_:27/27	Mean CPM Mean Compression Depth Complete Recoil	1 day
Kim et al. [32]	2017	Asia	RCT	Songs [v. metronome]	average 102	+	+	nr	≥100 cpm	1	T_1-29_:27/25	Mean CPM Mean Compression Depth Complete Recoil	1 day
Kneba et al. [33]	2020	Europe	Non-Randomized Control Trial	Stayin' Alive	103	+	+	-	90-110 cpm	0.75	T_0_: 19/15 T_>30_: 18/12	Dichotomous In Range	62-78 days

Characteristics that were cataloged included: continent of origin, study type, song title and beats per minute (BPM), population description (medically trained; Children), population, and sample size. We also identified if the integration of song was for instruction/coaching (active feedback) purposes or listening while performing (recall). Finally, we cataloged the target rate of intervention, duration of the test (Min), intervention/ control size, outcomes, and recall time periods (see Table 1).

Data items of interest included: percentage at adequate rate, variability of CPM, percentage with adequate depth, and variability compression depth.

Results

Study Selection

**Figure 1:**
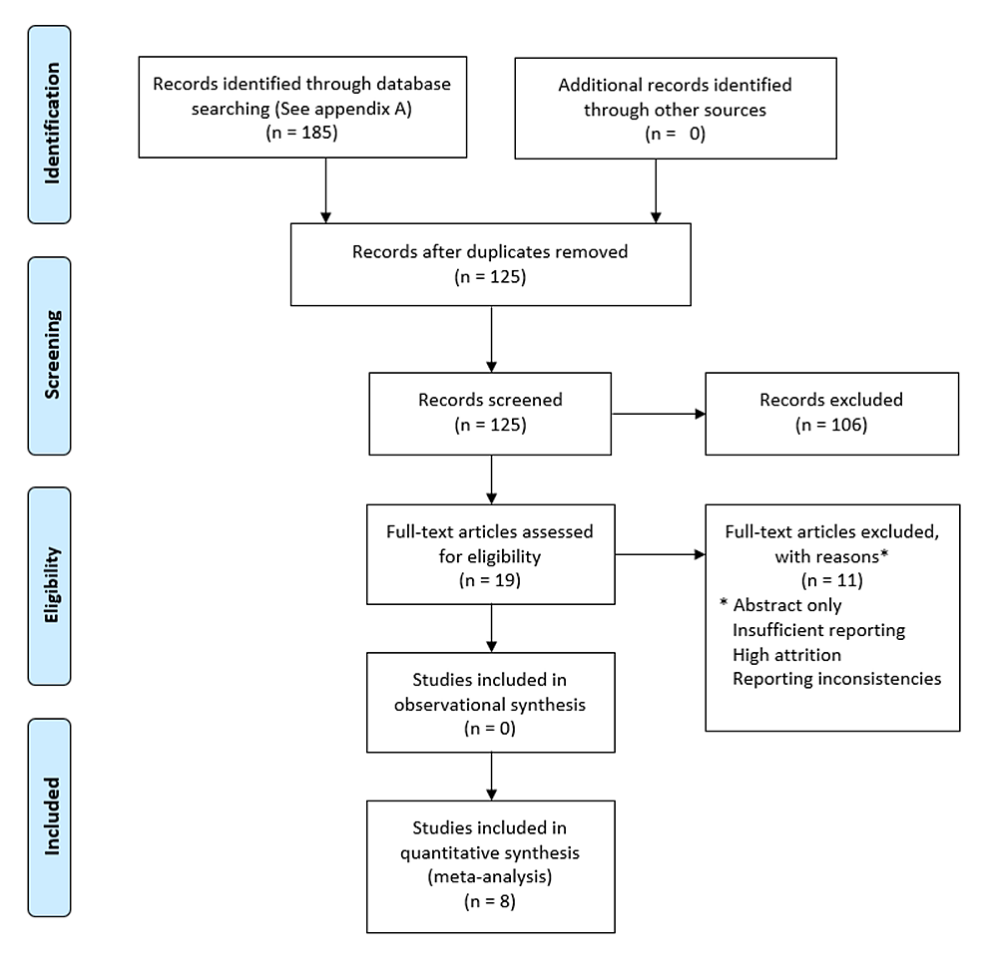
PRISMA Reporting From:  Moher D, Liberati A, Tetzlaff J, Altman DG, The PRISMA Group (2009). Preferred Reporting Items for Systematic Reviews and Meta-Analyses: The PRISMA Statement. PLoS Med 6(6): e1000097. doi:10.1371/journal.pmed1000097

Comprehensively, the search identified 125 unique citations (from results in PubMed-55; Embase-91; CINHAL-34; ERIC 1; Psychology and Behavioral Sciences Collection-1; Health Source: Nursing/Academic Edition / Health Source: Consumer Edition-2; and The Networked Digital Library of Theses and Dissertations (NDLTD)-2). Overall, eight studies were included for review. Common reasons for exclusion were the lack of a comparator or incomplete data from conference abstracts. For abstract only publications originally identified, we attempted contact with corresponding authors but received no additional information (see Figure 1).

Study Characteristics

The studies originated from multiple continents, including Asia (3), Australia (1), Europe (1), Europe/Asia (1), and N. America (2). Six included medically trained participants and none reported enrolling children. Six utilized songs in instruction and/ or coaching of compressions. Only two utilized songs while performing compressions in assessments. No studies reported any patient outcomes. We summarized the type of settings, populations, and study designs for each group (see Table 1).

The report of outcomes varied between the studies regarding measuring CPM. Some reported mean and standard deviations, while others reported a percentage of those in the target range. Mean compression depth was also identified for meta-analysis. We grouped the studies by times of assessment post-intervention with T_0_ representing immediately post-intervention, and then T_1_-_29 _or T_>30_ representing days post-intervention. Studies did present secondary outcomes but not in a manner for meta-analysis. The data are reported in individual study descriptions.

Critical Appraisal Within Studies

**Figure 2:**
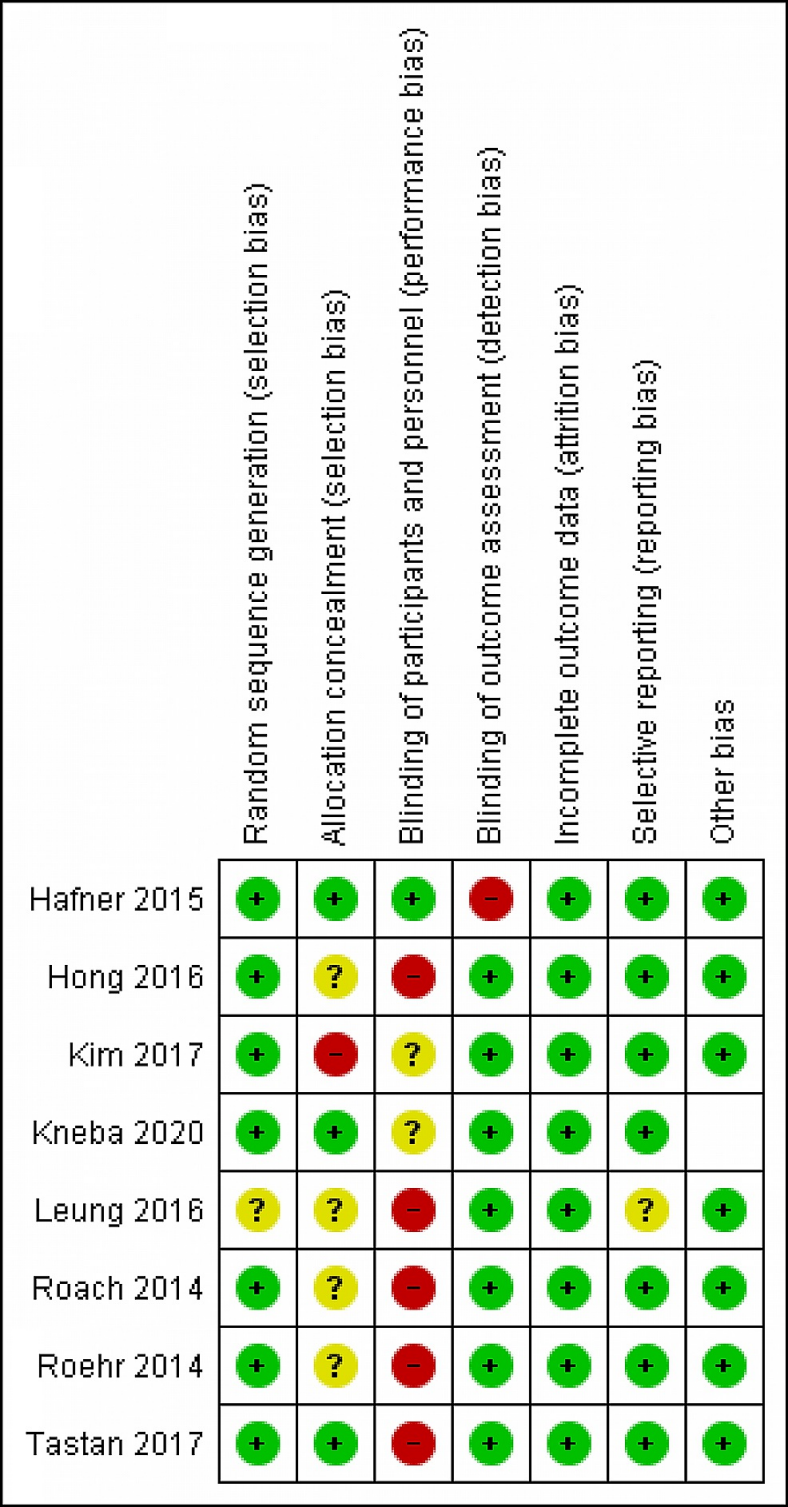
Risk of bias summary: review authors' judgments about each risk of bias item for each included study.

**Figure 3:**
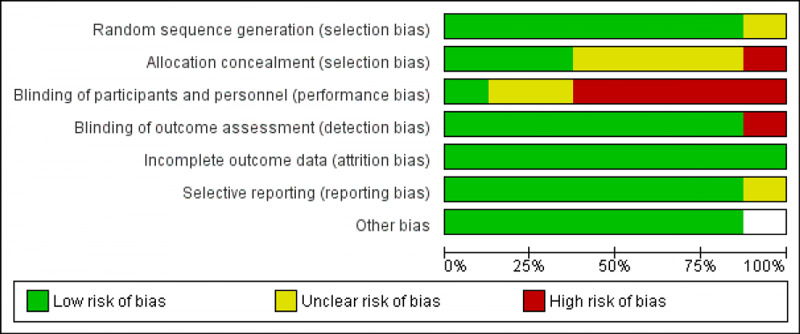
Risk of bias graph: review authors' judgments about each risk of bias item presented as percentage

Author B evaluated each included study for risk of bias, as recommended by the Preferred Reporting Items for Systematic Reviews and Meta-Analyses Extension for Scoping Reviews (PRISMA-ScR) Checklist [34]. Evaluation within the studies demonstrates a high-risk bias in terms of design and methodology (see Figures 2, 3).

 

Roach et al. worked with a group of nurses who did not perform at the recommended compression rate on a pre-test [26], nurses were randomized and retrained with the aid of coaching, demonstration, and the use of one of six songs chosen by the participant, compared to another group receiving similar retraining but without a song. It was noted that groups differed in variability ranging from 30-180 cpm in the no-song group compared to 80-150 cpm in the song group. At T_0_, there was a difference in favor of the song group averaging within the recommended rate, while the non-song group was just below rate, but significantly different in a ratio within the recommended range. At T_>30_ no difference was seen between groups and both performed within the recommended range or mean rate (see Tables 2, 3). Knowledge and confidence measures were not independent by the intervention group.

**Table 2:** Dichotomous outcomes of song group compared to other or no-song group within expected compression rate range Analysis computed by Review Manager (RevMan) [Computer program]. Version 5.3. Copenhagen: The Nordic Cochrane Centre, The Cochrane Collaboration, 2014.

	Song Group		Comparison Group						
Name	Within Range	Total 1	Within Range	Total 2	Effect Estimate	SE	CI Start	CI End	Weight
T_0_									
Leung [30]	13	17	17	20	0.6	0.8	0.1	3.0	6.0
Hafner et. al. [28]	24	50	18	46	1.4	0.4	0.6	3.2	10.3
Roach et al. [26]	21	32	13	32	2.8	0.5	1.0	7.7	9.2
Hong et al. [29]	65	68	53	61	3.3	0.7	0.8	12.9	7.3
Kneba et al. [33]	15	19	1	15	52.5	1.2	5.2	528.5	4.0
T_1-29_									
Kim et al. [32]	18	27	16	27	1.4	0.6	0.5	4.2	8.6
Leung [30]	15	17	16	20	1.9	0.9	0.3	11.8	5.4
Tastanet al. [31]	34	39	16	38	9.4	0.6	3.0	29.2	8.5
T_>30_	155	198	82	184	4.9		2.2	10.9	40.8
Roach et al. [26]	18	29	15	31	1.7	0.5	0.6	4.9	9.1
Hafner et al. [28]	34	46	18	42	3.8	0.5	1.5	9.3	9.8
Tastan et al. [31]	32	37	23	38	4.2	0.6	1.3	13.1	8.4
Kneba et al. [33]	9	18	1	12	11.0	1.1	1.2	103.9	4.1
Hong et al. [29]	62	68	25	61	14.9	0.5	5.6	39.7	9.3

**Table 3:** Mean compression rate outcomes of song group compared to other or no-song group Analysis computed by Review Manager (RevMan) [Computer program]. Version 5.3. Copenhagen: The Nordic Cochrane Centre, The Cochrane Collaboration, 2014.

	Song Group			Comparison Group							
	Mean	SD	Total	Mean	SD	Total	Mean Difference	Effect Estimate	SE	CI Start	CI End
T_0_											
Roach et al. [26]	106.7	9.0	32.0	99.1	9.0	32.0	7.6	2.3	3.2	12.0	15.4
Leung [30]	111.0	10.2	17.0	111.9	9.6	20.0	-0.9	3.3	-7.3	5.5	7.3
Hafner et al. [28]	109.0	15.0	50.0	121.0	21.0	46.0	-12.0	3.8	-19.4	-4.6	5.5
T_1-29_											
Leung [30]	107.9	5.0	17.0	108.1	8.3	20.0	-0.2	2.2	-4.5	4.1	15.9
Kim et al. [32]	98.2	9.1	27.0	98.5	16.7	25.0	-0.3	3.8	-7.7	7.1	5.5
Kim et al. [32]	98.2	9.1	27.0	110.4	15.3	27.0	-12.2	3.4	-18.9	-5.5	6.6
Tastan et al. [31]	107.3	7.3	39.0	121.5	12.9	38.0	-14.1	2.4	-18.8	-9.4	13.6
T_>30_											
Tastan et al. [31]	106.2	8.7	37.0	100.7	9.5	38.0	5.5	2.1	1.4	9.7	17.5
Roach et al. [26]	103.9	14.0	29.0	100.9	12.0	31.0	3.0	3.4	-3.6	9.6	6.8
Hafner et al. [28]	111.0	13.0	46.0	120.0	20.0	42.0	-9.0	3.6	-16.1	-1.9	5.9

Roehr et al. [27] compared six simulations of infant resuscitation each with a different song (randomized from a group of five) and no-song (baseline). Thirty medical professionals participated in the study, working in teams of two, alternating compressions and inflation by song. Of the five songs, only Abba's song “SOS," [35] was significantly different than the baseline and closer to the intended rate, for compressions and ventilations. Two of the comparison songs were also at 120-bpm as SOS but not statistically different from the other comparisons. This study was excluded from the meta-analysis because they included ventilation in its ratio and rate.

Hafner et al. [28] compared adults with no previous CPR training, one group who learned compression rate with the aid of a song at 103-bpm versus a control (no-song) group. The two groups were significantly different with the control group having a faster mean compression rate and a larger standard deviation at T_0_, 121 (SD 21) compared to 109 (SD 15). The odds ratio had a positive association toward the song group, but not significant. At T_>30_, the song group was closer to the target and the standard deviations remained wider in the no-song group. The odds ratio significantly favored songs for being in the recommended range. (See Tables 2-3.)

Hong et al. [29] compared adults without current CPR training, one group learned compression rate with the aid of a popular song in Korea versus a control group (metronome guided). Although there were no reported significant differences in the proportions with correct compression rate range, it was noted the popular song group performed at a significantly faster rate than the control group (107.4 cpm v. 102.2 cpm) at T_0_. However, at follow-up, the metronome group was significantly faster and on average faster than the recommended rate (124.8 cpm v. 110.0 cpm). This was reflected by a significant difference between the proportion of those in the recommended range for the popular song group (see Tables 2, 3). Hong et al. reported important outcomes of compression depth, the ratio of correct compression depth, and correct hand position were not significant between groups.

Leung [30] compared pediatric nurses retraining in infant CPR between those practicing with a song (103-bpm) and no-song. There were no significant differences in mean rate or proportion at the recommended rate (≥100 cpm) at T_0_. This study used a recall period of approximately one-week post-course; however, they also did a 30-minute practice session prior to this assessment, which was unique to this study. No significant difference in mean or proportion in the recommended rate was reported (see Tables 2, 3).

Tastan [31] compared student nurses initially learning CPR between those who practiced with a song (103-bpm) that was modified with an extra rhythm instrument - the traditional Turkish *dar-buka* and no-song. There were significant differences in mean rate one day after training, with the control group averaging 121.5 cpm compared to 107.3 cpm. The proportion in the recommended range also favored the song group. Six weeks post-training, the groups were more similar and both on average within the recommended range. However, the average and proportion of those in the recommended range were significantly different and supportive of the intervention (see Tables 2, 3).

Kim [32] compared third-year medical students compression rates between those who practiced with one of five songs (average bpm 102) from a pre-determined list to allow for familiarity, practiced with a metronome, or no-song. The assessment occurred 24 hours after practice. The mean compression rates were significantly different between the no-song and song group, with the song group just below the recommended rate (98.2 cpm) and the no-song in the recommended rate (110.4 cpm). The mean difference was not significantly different between the song and metronome groups, and both were below the recommend ≥100 cpm (see Tables 2, 3).

Kneba and Humm [33] compared students in a veterinary graduate program on compression rate on canine models between those who practiced with a song (103-bpm) or no-song. The mean compression rates were calculated but did not have standard deviations reported, which prevented meta-analysis. Only one participant of 15 in the no-song group achieved the 90-110 cpm expectation compared to 15 of 19 in the song group, a significant difference at T_0_. Similarly, at T_>30_ only one person in the no-song group compared to 8 of 18 in the song group were in the expected range. The average compression rate for the no-song group at both assessments exceeded the 110 cpm expectation (see Table 1).

Summary Findings

We utilized the GRADEpro Guideline Development Tool [Software] [36] and Review Manger 5.3 [37] to systematically analyze similar outcomes. For the critical outcome of compression rate, a meta-analysis of 446 song-group participants compared to 443 no-song group participants yielded an odds ratio (OR) of 3.47 (confidence interval [CI] 2.00, 6.03) favouring the song group (see Appendix B). At specific time categories, odds ratios changed but all favored songs. At T_0 _the odds ratio favors the use of songs, however, the confidence interval does not communicate independence from the no-song group (OR 2.60, CI 0.97, 6.97). The mean compression rates were not statistically different (mean difference 1.56, CI -1.70, 4.82). And at T_1-29 _the odds ratio again favored the use of songs but did not show independence from the non-song group (OR 3.02, CI 0.81, 11.28). The mean compression rates were independent, where the song group on average was slower (mean difference -6.68, CI -9.37, -4.00). Then again at T_>30 _the odds ratio favored the use of songs and showed independence from the non-song group at >30 days (OR 4.92, CI 2.22, 10.94). The mean compression rates were not statistically different (mean difference 2.12, CI -1.02, 5.27).

For the critical outcome of compression depth, five studies reported mean depth [26,28,30-32]. From a meta-analysis of the combined studies, the mean differences at T_0_, T_1-29_, and T_>30_ were not significant or when combined (see Appendix C). With one exception, the mean differences showed a greater propensity and proximity to the guideline recommendation of a minimum of 50 mm in the non-song group. All studies showed an average depth shallower than the recommendations.

For the important outcomes, chest compression fraction, learner self-efficacy, and learner confidence, there were not consistently used measures between studies. Roach et al. [26] did not identify any significant difference in learner knowledge or confidence in the intervention group. Hong et al. [29] did not identify any significant differences in learner ratio of correct compression depth, or correct hand position. Leung [30] did not identify any significant differences in learner outcomes of complete recoil or no flow times. Kim et al. [32] did not find any significant difference in complete recoil between song, metronome, or no-song groups.

Summary of Evidence

There is limited evidence to make any standard for the use of a specific song or songs in general because of risk of biases and inconsistency of data reporting, however, trends of outcomes provide educators and training organizations direction, from which they may choose to act. For the outcome of greater consistency (aka, smaller standard deviations) the use of songs can contribute to learning outcomes and should be considered a tool for instructors. The choice of song based on BPM has not been established as to where an ideal rate would fall in the given 100-120 range. Notably, for the song group, no average CPM rate was >110, which corresponded with the lower BPM of songs used in the studies. This leaves the need to assess the effect of faster BPM songs on rate and depth to identify value or costs. It is important to emphasize that six of the eight studies were with medically trained participants leaving less direct evidence for the use of songs with the training of lay responders; however, differences in outcomes were not noted. 

Depth for all studies was on average shallower for the song groups, which may be the result of attention to rate versus depth or a physiological limitation of rate on depth. There may be some cognitive load on learners, imparted during the studies (bias), particularly in song groups, to emphasize rate. For example, students were encouraged to recall songs during assessments. Future studies may seek to develop an educational emphasis on depth, using songs as a mental metronome to limit cognitive load.

To improve future evidence development and systematic reviews, we encourage that data be reported in dichotomous and continuous formats. Minimum reporting categories for original research moving forward to enhance future comparisons should be established. We recommend that a specific segment of the song used to be analyzed for BPM for consistent and accurate reporting, as there are fluctuations of tempo throughout songs and entire songs are not used. Reporting the role or intention of the song in training (e.g., background beat, synchronization of practice compressions, synchronization during testing) would allow for better comparison and understanding in educational practice. Participant demographics should include if learners were new or previously trained in CPR. Outcomes should be predicated on a stated hypothesis, which was not consistently reported. We also recommend that testing duration be a minimum two minutes to mimic the expectation of five cycles of CPR and when it is more natural to switch compressors. For outcome reporting, average CPM by minute and average compression depth by minute would better describe the role of the intervention in maintaining consistency. Finally, we recommend that authors utilize the Guideline for Reporting Evidence-Based practice Educational interventions and Teaching (GREET) to enhance consistency and validity during the research process.

We believe there is an opportunity for future research to establish the theory and practice of using songs for educational use in CPR. Foundationally, the field must establish that their population/sample can follow a beat, as potentially 14% of the population may have amusia (aka beat deafness) [38], and be able to compress deep enough at the recommended rate prior to assessing song compliance. Then, future studies on the use of songs must communicate if the song(s) used is already known to or learned by participants versus an unknown/unfamiliar song, as this can impact the length of training and longitudinal recall. Once the previous assumptions are examined, future research between medically trained and lay responders would acknowledge any differences in pedagogy needed within those groups. Additionally, the research could be done between lay responder populations, for example, their use with children. Practically, there is no consensus on what BPM of a song/song segment produces ideal cadence, which needs to be a priority research topic. And, as Tastan et al. [31] brought up, can songs have their beats enhanced to influence results?

An additional area for exploration would be in comparing active compression feedback devices and songs for learning appropriate rate. Active feedback devices offer different interventions to improve compression performance. However future studies can compare outcomes to financial costs of purchasing, maintenance, time on task, etc. of learners and educators between the two tools.

Limitations

The original question wanted to include outcomes specific to lay responders due to their unique position of initially learning CPR without ongoing support or testing. The body of literature did not allow us to discriminate between lay responders and medically trained responders. Extrapolation between groups may be appropriate as they had similar outcomes in this review, but future research is needed to identify specific characteristics of songs that may prove useful to recalling and performing effective CPR compressions. These future explorations may also include socio-cultural elements of specific songs or style of songs [29], for example, the use of instruments to accent the beat [31]. Similarly, the lack of studies from around the globe may limit the generalization of outcomes based on cultural nuances.

The 20-bpm range difference in lower and upper thresholds of the current recommendations makes comparisons for any clinical differences challenging. The literature does not suggest that 1-bpm over or under is detrimental, nor that compressing at 110-bpm is better than 100-bpm or 120-bpm. The Revman5 software had limited ability to deal with reported means when reported within a range versus a single figure. The reports of BPM by song are somewhat problematic as a full song varies in BPM during different sections in some cases. Only one study [29] reported the BPM for the segment used in their study, while others did not differentiate the whole song or segment.

## Conclusions

From an educational perspective, there is value to the introduction and use of songs for the purpose of narrowing the deviation from recommended ranges and increasing the odds of being within a targeted range during CPR skill education. Longitudinal recall appears to be also favored within groups using songs, which supports the need for additional research as to how, why, and which types of songs would be effective. However, the ideal song is not described in the literature regarding qualities of cultural relevance, distinct cadence, or BPM. We suggest that instructors be able to identify songs that resonate with learners and have a BPM of approximately 110 bpm to better meet recommended compression rate range. More evidence is needed regarding the effect of this on compression depth and recoil as well as the influence of songs on confidence or willingness to perform CPR.

## Appendices

Appendix A: Search Strategy

PubMed (New Pubmed)

([cpr] OR ["chest compression*"]) OR ("chest wall oscillation"[MeSH Terms] OR "chest wall oscillation"[All Fields]) AND ([music] OR [singing]) OR ([song*]) OR ([metronome*]) NOT (Song[Author]) Filters: from 2014 to 2020 

Search Details:

(((((("cardiopulmonary resuscitation"[MeSH Terms] OR ("cardiopulmonary"[All Fields] AND "resuscitation"[All Fields])) OR "cardiopulmonary resuscitation"[All Fields]) OR "cpr"[All Fields]) OR "chest compression*"[All Fields]) OR ("chest wall oscillation"[MeSH Terms] OR "chest wall oscillation"[All Fields])) AND (((((((((("music"[MeSH Terms] OR "music"[All Fields]) OR "music's"[All Fields]) OR "musical"[All Fields]) OR "musicality"[All Fields]) OR "musically"[All Fields]) OR "musicals"[All Fields]) OR "musics"[All Fields]) OR ("singing"[MeSH Terms] OR "singing"[All Fields])) OR "song*"[All Fields]) OR "metronome*"[All Fields])) NOT Song[Author]

55 Results, January 22, 2020

**Table 4:** Embase search queries

No.	Query	Results	Date
#6	(((('resuscitation'/exp OR 'cardiopulmonary resuscitation'/exp OR 'cardio pulmonary resuscitation'/exp OR 'cardiopulmonary resuscitation':ab,ti OR 'cardio pulmonary resuscitation':ab,ti OR cpr:ab,ti OR 'bystander cpr':ab,ti OR 'chest compression':ab,ti OR 'cardiac compression':ab,ti OR 'chest wall compression':ab,ti OR 'thoracic compression':ab,ti OR 'cardiac wall compression':ab,ti OR 'thoracic wall compression':ab,ti OR 'hands-only resuscitation':ab,ti OR 'hands only resuscitation':ab,ti OR 'compression-only cpr':ab,ti OR 'compression only cpr':ab,ti OR 'heart massage':ab,ti OR 'cardiac massage':ab,ti OR 'thoracic massage':ab,ti OR 'heart arrest/therapy' OR 'cardiac arrest/therapy' OR 'thoracic arrest/therapy') AND ('music'/exp OR music:ab,ti OR song:ab,ti OR songs:ab,ti OR 'singing'/exp OR singing:ab,ti OR humming:ab,ti OR tune:ab,ti OR tunes:ab,ti OR 'melody'/exp OR melody:ab,ti OR melodies:ab,ti OR 'metronome'/exp OR metronome:ab,ti OR metronomes:ab,ti)) AND (2014:py OR 2015:py OR 2016:py OR 2017:py OR 2018:py OR 2019:py OR 2020:py)) NOT 'melody valve') NOT song:au	91	Jan 22, 2020
#5	((('resuscitation'/exp OR 'cardiopulmonary resuscitation'/exp OR 'cardio pulmonary resuscitation'/exp OR 'cardiopulmonary resuscitation':ab,ti OR 'cardio pulmonary resuscitation':ab,ti OR cpr:ab,ti OR 'bystander cpr':ab,ti OR 'chest compression':ab,ti OR 'cardiac compression':ab,ti OR 'chest wall compression':ab,ti OR 'thoracic compression':ab,ti OR 'cardiac wall compression':ab,ti OR 'thoracic wall compression':ab,ti OR 'hands-only resuscitation':ab,ti OR 'hands only resuscitation':ab,ti OR 'compression-only cpr':ab,ti OR 'compression only cpr':ab,ti OR 'heart massage':ab,ti OR 'cardiac massage':ab,ti OR 'thoracic massage':ab,ti OR 'heart arrest/therapy' OR 'cardiac arrest/therapy' OR 'thoracic arrest/therapy') AND ('music'/exp OR music:ab,ti OR song:ab,ti OR songs:ab,ti OR 'singing'/exp OR singing:ab,ti OR humming:ab,ti OR tune:ab,ti OR tunes:ab,ti OR 'melody'/exp OR melody:ab,ti OR melodies:ab,ti OR 'metronome'/exp OR metronome:ab,ti OR metronomes:ab,ti)) AND (2014:py OR 2015:py OR 2016:py OR 2017:py OR 2018:py OR 2019:py OR 2020:py)) NOT 'melody valve'	91	Jan 22, 2020
#4	(('resuscitation'/exp OR 'cardiopulmonary resuscitation'/exp OR 'cardio pulmonary resuscitation'/exp OR 'cardiopulmonary resuscitation':ab,ti OR 'cardio pulmonary resuscitation':ab,ti OR cpr:ab,ti OR 'bystander cpr':ab,ti OR 'chest compression':ab,ti OR 'cardiac compression':ab,ti OR 'chest wall compression':ab,ti OR 'thoracic compression':ab,ti OR 'cardiac wall compression':ab,ti OR 'thoracic wall compression':ab,ti OR 'hands-only resuscitation':ab,ti OR 'hands only resuscitation':ab,ti OR 'compression-only cpr':ab,ti OR 'compression only cpr':ab,ti OR 'heart massage':ab,ti OR 'cardiac massage':ab,ti OR 'thoracic massage':ab,ti OR 'heart arrest/therapy' OR 'cardiac arrest/therapy' OR 'thoracic arrest/therapy') AND ('music'/exp OR music:ab,ti OR song:ab,ti OR songs:ab,ti OR 'singing'/exp OR singing:ab,ti OR humming:ab,ti OR tune:ab,ti OR tunes:ab,ti OR 'melody'/exp OR melody:ab,ti OR melodies:ab,ti OR 'metronome'/exp OR metronome:ab,ti OR metronomes:ab,ti)) AND (2014:py OR 2015:py OR 2016:py OR 2017:py OR 2018:py OR 2019:py OR 2020:py)	95	Jan 22, 2020
#3	('resuscitation'/exp OR 'cardiopulmonary resuscitation'/exp OR 'cardio pulmonary resuscitation'/exp OR 'cardiopulmonary resuscitation':ab,ti OR 'cardio pulmonary resuscitation':ab,ti OR cpr:ab,ti OR 'bystander cpr':ab,ti OR 'chest compression':ab,ti OR 'cardiac compression':ab,ti OR 'chest wall compression':ab,ti OR 'thoracic compression':ab,ti OR 'cardiac wall compression':ab,ti OR 'thoracic wall compression':ab,ti OR 'hands-only resuscitation':ab,ti OR 'hands only resuscitation':ab,ti OR 'compression-only cpr':ab,ti OR 'compression only cpr':ab,ti OR 'heart massage':ab,ti OR 'cardiac massage':ab,ti OR 'thoracic massage':ab,ti OR 'heart arrest/therapy' OR 'cardiac arrest/therapy' OR 'thoracic arrest/therapy') AND ('music'/exp OR music:ab,ti OR song:ab,ti OR songs:ab,ti OR 'singing'/exp OR singing:ab,ti OR humming:ab,ti OR tune:ab,ti OR tunes:ab,ti OR 'melody'/exp OR melody:ab,ti OR melodies:ab,ti OR 'metronome'/exp OR metronome:ab,ti OR metronomes:ab,ti)	185	Jan 22, 2020
#2	'music'/exp OR music:ab,ti OR song:ab,ti OR songs:ab,ti OR 'singing'/exp OR singing:ab,ti OR humming:ab,ti OR tune:ab,ti OR tunes:ab,ti OR 'melody'/exp OR melody:ab,ti OR melodies:ab,ti OR 'metronome'/exp OR metronome:ab,ti OR metronomes:ab,ti	55,168	Jan 22, 2020
#1	'resuscitation'/exp OR 'cardiopulmonary resuscitation'/exp OR 'cardio pulmonary resuscitation'/exp OR 'cardiopulmonary resuscitation':ab,ti OR 'cardio pulmonary resuscitation':ab,ti OR cpr:ab,ti OR 'bystander cpr':ab,ti OR 'chest compression':ab,ti OR 'cardiac compression':ab,ti OR 'chest wall compression':ab,ti OR 'thoracic compression':ab,ti OR 'cardiac wall compression':ab,ti OR 'thoracic wall compression':ab,ti OR 'hands-only resuscitation':ab,ti OR 'hands only resuscitation':ab,ti OR 'compression-only cpr':ab,ti OR 'compression only cpr':ab,ti OR 'heart massage':ab,ti OR 'cardiac massage':ab,ti OR 'thoracic massage':ab,ti OR 'heart arrest/therapy' OR 'cardiac arrest/therapy' OR 'thoracic arrest/therapy'	122,839	Jan 22, 2020

**Table 5:** CINAHL search queries

Wednesday, January 22, 2020, 12:20:33 PM				
#	Query	Limiters/Expanders	Last Run Via	Results
S3	S1 AND S2	Limiters - Published Date: 20140101-20201231 Expanders - Also search within the full text of the articles Search modes - Boolean/Phrase	Interface - EBSCOhost Research Databases Search Screen - Advanced Search Database - CINAHL with Full Text	34
S2	(AB (music OR song OR songs OR singing OR hum OR humming OR tune OR tunes OR melody OR melodies OR metronome OR metronomes) OR TI (music OR song OR songs OR singing OR hum OR humming OR tune OR tunes OR melody OR melodies OR metronome OR metronomes) OR (MH "Music") OR (MH "Singing"))	Expanders - Also search within the full text of the articles Search modes - Boolean/Phrase	Interface - EBSCOhost Research Databases Search Screen - Advanced Search Database - CINAHL with Full Text	19,459
S1	(AB (“cardiopulmonary resuscitation” OR “cardio pulmonary resuscitation” OR cpr OR “bystander cpr” OR “chest compression” OR “cardiac compression” OR “chest wall compression” OR “thoracic compression” OR “cardiac wall compression” OR “thoracic wall compression” OR “hands-only resuscitation” OR “hands only resuscitation” OR “compression-only cpr” OR “compression only cpr” OR “heart massage” OR “cardiac massage” OR “thoracic massage” OR ("heart arrest" AND therapy) OR ("cardiac arrest" AND therapy) OR ("thoracic arrest" AND therapy)) OR TI (“cardiopulmonary resuscitation” OR “cardio pulmonary resuscitation” OR cpr OR “bystander cpr” OR “chest compression” OR “cardiac compression” OR “chest wall compression” OR “thoracic compression” OR “cardiac wall compression” OR “thoracic wall compression” OR “hands-only resuscitation” OR “hands only resuscitation” OR “compression-only cpr” OR “compression only cpr” OR “heart massage” OR “cardiac massage” OR “thoracic massage” OR ("heart arrest" AND therapy) OR ("cardiac arrest" AND therapy) OR ("thoracic arrest" AND therapy)) OR (MH "Resuscitation, Cardiopulmonary") OR (MH "Bystander CPR") OR (MH "Heart Massage") OR (MH "Heart Arrest/TH"))	Expanders - Also search within the full text of the articles Search modes - Boolean/Phrase	Interface - EBSCOhost Research Databases Search Screen - Advanced Search Database - CINAHL with Full Tex	

ERIC Database

Date Searched: January 22, 2020

("Cardiopulmonary Resuscitation" OR CPR) AND (music OR song OR sing OR metronome OR hum OR tune OR melody)

1 Result - Not relevant because it was outside of the specified date range.

**Table 6:** Psychology and Behavioral Sciences Collection Database Searched Wednesday, January 22, 2020 12:09:28 PM

#	Query	Limiters/Expanders	Last Run Via	Results
S3	S1 AND S2	Limiters - Published Date: 20140101-20201231 Expanders - Also search within the full text of the articles Search modes - Boolean/Phrase	Interface - EBSCOhost Research Databases Search Screen - Advanced Search Database - Psychology and Behavioral Sciences Collection	1
S2	(DE "MUSIC" OR DE "MUSIC in education" OR DE "SONGS" OR DE "MELODY" OR DE "MUSICAL composition" OR AB (music OR song OR songs OR singing OR hum OR humming OR tune OR tunes OR melody OR melodies OR metronome OR metronomes) OR TI (music OR song OR songs OR singing OR hum OR humming OR tune OR tunes OR melody OR melodies OR metronome OR metronomes))	Expanders - Also search within the full text of the articles Search modes - Boolean/Phrase	Interface - EBSCOhost Research Databases Search Screen - Advanced Search Database - Psychology and Behavioral Sciences Collection	6,960
S1	(DE "CPR (First aid)" OR DE "CARDIAC arrest" OR DE "CARDIAC massage" OR DE "CARDIAC resuscitation" OR DE "BYSTANDER CPR" OR AB (“cardiopulmonary resuscitation” OR “cardio pulmonary resuscitation” OR cpr OR “bystander cpr” OR “chest compression” OR “cardiac compression” OR “chest wall compression” OR “thoracic compression” OR “cardiac wall compression” OR “thoracic wall compression” OR “hands-only resuscitation” OR “hands only resuscitation” OR “compression-only cpr” OR “compression only cpr” OR “heart massage” OR “cardiac massage” OR “thoracic massage” OR ("heart arrest" AND therapy) OR ("cardiac arrest" AND therapy) OR ("thoracic arrest" AND therapy)) OR TI (“cardiopulmonary resuscitation” OR “cardio pulmonary resuscitation” OR cpr OR “bystander cpr” OR “chest compression” OR “cardiac compression” OR “chest wall compression” OR “thoracic compression” OR “cardiac wall compression” OR “thoracic wall compression” OR “hands-only resuscitation” OR “hands only resuscitation” OR “compression-only cpr” OR “compression only cpr” OR “heart massage” OR “cardiac massage” OR “thoracic massage” OR ("heart arrest" AND therapy) OR ("cardiac arrest" AND therapy) OR ("thoracic arrest" AND therapy)))	Expanders - Also search within the full text of the articles Search modes - Boolean/Phrase	Interface - EBSCOhost Research Databases Search Screen - Advanced Search Database - Psychology and Behavioral Sciences Collection	875

**Table 7:** PsycARTICLES Search Queries Searched Wednesday, January 22, 2020 3:20:33 PM

#	Query	Limiters/Expanders	Last Run Via	Results
S3	S1 AND S2	Limiters - Year of Publication: 2014-2020 Expanders - Apply equivalent subjects Search modes - Boolean/Phrase	Interface - EBSCOhost Research Databases Search Screen - Advanced Search Database - APA PsycArticles	0
S2	music OR musical OR singing OR song OR songs OR prompt* OR metronome*	Expanders - Apply equivalent subjects Search modes - Boolean/Phrase	Interface - EBSCOhost Research Databases Search Screen - Advanced Search Database - APA PsycArticles	3,795
S1	CPR OR cardiopulmonary resuscitation OR cardiorespiratory resuscitation OR chest compressions OR cardiac arrest	Expanders - Apply equivalent subjects Search modes - Boolean/Phrase	Interface - EBSCOhost Research Databases Search Screen - Advanced Search Database - APA PsycArticles	19

**Table 8:** Health Source: Nursing/Academic Edition/Health Source: Consumer Edition Search Queries Searched Wednesday, January 22, 2020, 2:04:54 PM

#	Query	Limiters/Expanders	Last Run Via	Results
S5	s3 NOT s4	Limiters - Scholarly (Peer Reviewed) Journals; Published Date: 20140101-20181231 Expanders - Apply equivalent subjects Search modes - Boolean/Phrase	Interface - EBSCOhost Research Databases Search Screen - Advanced Search Database - Health Source: Nursing/Academic Edition;Health Source - Consumer Edition	2
S4	AU song	Expanders - Apply equivalent subjects Search modes - Boolean/Phrase	Interface - EBSCOhost Research Databases Search Screen - Advanced Search Database - Health Source: Nursing/Academic Edition;Health Source - Consumer Edition	5,363
S3	(DE "CPR (First aid)" OR DE "CPR education" OR (cpr or cardiopulmonary resuscitation or cardiorespiratory resuscitation or chest compressions or cardiac arrest)) AND (S1 AND S2)	Expanders - Apply equivalent subjects Search modes - Boolean/Phrase	Interface - EBSCOhost Research Databases Search Screen - Advanced Search Database - Health Source: Nursing/Academic Edition;Health Source - Consumer Edition	38
S2	(DE "CPR (First aid)" OR DE "CPR education”) OR (cpr or cardiopulmonary resuscitation or cardiorespiratory resuscitation or chest compressions or cardiac arrest)	Expanders - Apply equivalent subjects Search modes - Boolean/Phrase	Interface - EBSCOhost Research Databases Search Screen - Advanced Search Database - Health Source: Nursing/Academic Edition;Health Source - Consumer Edition	7,742
S1	(music OR song OR songs OR musical) OR (DE "MELODY" OR DE "MUSIC in education" OR DE "MUSIC psychology" OR DE "SONGS”)	Expanders - Apply equivalent subjects Search modes - Boolean/Phrase	Interface - EBSCOhost Research Databases Search Screen - Advanced Search Database - Health Source: Nursing/Academic Edition;Health Source - Consumer Edition	21,919

The Networked Digital Library of Theses and Dissertations (NDLTD)

Date Searched: January 22, 2020

description: ("cardiopulmonary resuscitation" OR CPR OR "chest compression" OR "heart massage") AND description: (music OR song OR songs OR singing OR hum OR humming OR tunes OR melody OR melodies OR metronome OR metronomes)

Filters: Publication year 2014-2020

1 result

Appendix B: GRADE Evidence Profile of Dichotomous Outcomes of Compression Rates Within Expected Range and Mean Compression Rate

**Table 9:** GRADE evidence profile of dichotomous outcomes of compression rates within expected range and mean compression rate ^a^No blinding. Convenience populations; ^b^reporting of outcomes

Certainty assessment							No. of patients		Effect		Certainty	Importance
No. of studies	Study design	Risk of bias	Inconsistency	Indirectness	Imprecision	Other considerations	Song	No Song	Relative (95% CI)	Absolute (95% CI)		
Dichotomous												
7	randomized trials	very serious ^a^	serious ^b^	not serious	not serious	none	360/467 (77.1%)	232/443 (52.4%)	OR 3.47 (2.00 to 6.03)	269 more per 1,000 (from 164 more to 345 more)	⨁◯◯◯ VERY LOW	CRITICAL
Dichotomous - Day 0												
5	randomized trials	very serious ^a^	serious ^b^	not serious	not serious	none	138/186 (74.2%)	102/174 (58.6%)	OR 2.60 (0.97 to 6.97)	200 more per 1,000 (from 7 fewer to 322 more)	⨁◯◯◯ VERY LOW	CRITICAL
Dichotomous - Day 1-29												
3	randomised trials	very serious ^a^	serious ^b^	not serious	not serious	none	67/83 (80.7%)	48/85 (56.5%)	OR 3.02 (0.81 to 11.28)	232 more per 1,000 (from 52 fewer to 371 more)	⨁◯◯◯ VERY LOW	CRITICAL
Dichotomous - Days > 30												
5	randomised trials	very serious ^a^	serious ^b^	not serious	not serious	none	155/198 (78.3%)	82/184 (44.6%)	OR 4.92 (2.22 to 10.94)	353 more per 1,000 (from 195 more to 452 more)	⨁◯◯◯ VERY LOW	CRITICAL

Appendix C: GRADE Evidence Profile of Mean Compression Depth

**Table 10:** GRADE evidence profile of mean compression depth CI: Confidence interval; OR: Odds ratio; MD: Mean difference ^a^No blinding. Convenience populations.

Certainty assessment							No. of patients		Effect		Certainty	Importance
No. of studies	Study design	Risk of bias	Inconsistency	Indirectness	Imprecision	Other considerations	Song	No Song	Relative (95% CI)	Absolute (95% CI)		
Compression Depth												
4	randomised trials	serious ^a^	not serious	not serious	not serious		293	277	-	MD 0.64 lower (1.24 lower to 0.04 lower)	-	
Compression Depth - Day 0												
3	randomised trials	very serious ^a^	not serious	not serious	not serious		135	127	-	MD 0.9 lower (3.22 lower to 1.42 higher)	-	
Compression Depth - 1-29												
2	randomised trials	very serious ^a^	not serious	not serious	not serious		44	47	-	MD 0.55 lower (1.19 lower to 0.09 higher)	-	
Compression Depth - >30												
2	randomised trials	very serious ^a^	not serious	not serious	not serious		114	103	-	MD 2.11 lower (4.97 lower to 0.76 higher)	-	
